# *IL-10 (-819C/T), TNFA (-30G/A)* and *ENOS (-786T/C)* Polymorphisms Modulating the Outcome Related to Mental Disorders in Crack Addicted Users

**DOI:** 10.2174/17450179-v18-e2201140

**Published:** 2022-02-08

**Authors:** Ana Caroline Melo dos Santos, Barbara Rayssa Correia dos Santos, Bruna Brandão dos Santos, Edilson Leite de Moura, Abel Barbosa Lira Neto, Aline Cristine Pereira e Silva, Karol Fireman de Farias, Verônica de Medeiros Alves, Antônio Egídio Nardi, Elaine Virgínia Martins de Souza Figueiredo

**Affiliations:** 1 Program in Health Sciences, Molecular Biology and Gene Expression Laboratory, Federal University of Alagoas, Maceio, Brazil; 2 Laboratory of Molecular Chronobiology, Federal University of Alagoas, Maceio, Brazil; 3 Program in Nursing, Molecular Biology and Gene Expression Laboratory, Federal University of Alagoas, Maceio, Brazil; 4 Department of Nursing, Federal University of Alagoas, Maceio, Brazil; 5 Institute of Psychiatry, Federal University of Rio de Janeiro, Porto Alegre, Brazil; 6 Program in Health Sciences, Molecular Biology and Gene Expression Laboratory, Federal University of Alagoas, Maceio, Brazil

**Keywords:** Genetic polymorphism, Cocaine, Crack, Mental disorders, Central nervous system, Chronic depressive symptoms

## Abstract

**Background:**

Cocaine/crack use affects immune system molecules and development of mental disorders has been identified.

**Objective:**

To investigate the relationship of polymorphisms in the *TNFA* (-308G/A), IL-10 (-819C/T) and *ENOS* (-786T/C) genes with mental disorders in cocaine and crack users.

**Methods:**

A case-control study was carried out, which included 107 cocaine and crack users and 115 controls who never used healthy cocaine and crack. The SNPs in the *TNFA* (-308G/A), *IL-10* (-819C/T) and *ENOS* (-786T/C) genes were genotyped by real time PCR.

**Results:**

As for the individuals included in this study, the average age of 31.4 years (± 8.59). We identified that the G/A genotype to TNFA (-308) (OR = 0.24; p = 0.03) and the A allele (OR = 0.30; p = 0.03) were associated with reduced risk for dysthymic disorder. The T allele of the IL-10 (-819) polymorphism was associated with decreased risk of developing panic disorder (OR = 0.44; p = 0.01), while the C allele was correlated with an increased risk for alcohol dependence (OR = 1.97; p = 0.04), alcohol abuse (OR = 1.81; p = 0.04) and psychotic syndrome (OR = 2.23; p = 0.01). C/C genotype was correlated with increased chances of developing current psychotic syndrome (OR = 4.23; p = 0.01).

**Conclusion:**

Our results suggest that genetic polymorphisms promote susceptibility or promote protection for clinical phenotypes of psychiatric comorbidities in cocaine and crack users and be considered as good prognostic markers.

## 1 INTRODUCTION

Cocaine is used by an estimated 18.2 million people worldwide, about 0.4 percent of the global population aged between 15 and 64 years [1]. Disorders triggered by the use of cocaine and crack consist of a complex public health problem that involves consequences at the biological level and in social behavior [2, 3]. The chronic use of cocaine is often accompanied by several mental disorders, such as chronic depressive symptoms [4], suicide risk, post-traumatic stress disorder, antisocial personality disorder [5], and anxiety disorders [6].

Recent studies have shown that drug abuse has an immunomodulatory effect influencing the signaling and gene expression of the immune system [7, 8], and oxidative damage [9, 10]. Evidence suggests that chronic cocaine use changes the profile of plasma levels of cytokines and lymphocytes Th1/Th2/Th17, natural killer (NK) cells, and nitric oxide [7, 11-13], however the mechanism is unidentified. This evidence indicates that alterations in these profiles may serve as a relevant biomarker of an inflammatory state in the central nervous system. Thus, neuroinflammation has been proposed to be a mechanism that facilitates dopamine signaling in the brain [14] and inflammatory state induced by oxidative stress from cocaine, stimulates the hypothalamus-pituitary-adrenal (HPA) axis and changes cytokine levels [5], thereby leading to addicted behavior and susceptibility to mental disorders.

In a population of cocaine addicts in rehabilitation in the United States, a significant reduction in tumor necrosis factors α (TNF – alpha) was observed [15]; also, an *in vivo* study involving mice treated with cocaine revealed similar results [16], as well as an increase in the production of Interleukin (IL)-4 and Interleukin (IL)-10 [16, 17]. Moreover, in cocaine users with mental disorders, a significant difference in the cytokine profile was observed, suggesting that the plasma profile of cytokines can be used to monitor and stratify patients who are undergoing treatment for addiction, and thus, better manage the risk for the development of other psychiatric comorbidities [18]; however, there are no studies with a genetic approach.

From this perspective, several studies have demonstrated the role of single nucleotide polymorphisms (*SNPs*) in molecules of the immune system and oxidative stress that contribute to susceptibility to mental disorders [19-21]; however, there are no investigations involving drug users to understand the development of these disorders. In the present study, we hypothesize that *IL-10 (-819C/T), TNFA (-30G/A8)* and *ENOS (-786T/C)* polymorphisms can modulate the outcome of mental disorders in cocaine and crack users. The aim of this study was to investigate the role of genetic polymorphisms of cytokines and oxidative stress in the development of psychiatric comorbidities in cocaine and crack users.

## 2 METHODS

### 2.1 Participants

The study was approved by the Ethics Committee of the Federal University of Alagoas, and all participants provided a free and informed consent term (protocol nº 2.408.885 and CAAE 67643417.3.0000.5013). For this research, we conducted a case-control study with a sample of 107 cocaine and crack users recruited from three therapeutic community groups in a state in Northeast Brazil. The control group (n=115) was composed of healthy volunteers who did not report an established clinical diagnosis of psychiatric comorbidities, were without positive diagnoses according to the MINI International Neuropsychiatric Interview (MINI version 5.0), were 18 years old, who reported that they had never used cocaine and crack, and agreed to participate in the research.

The inclusion criteria involved individuals over 18 years old who met the criteria for addiction, according to the Diagnostic and Statistical Manual of Mental Disorders, 4th Edition (DSM-IV), and reported crack/cocaine use. The exclusion criteria included a prior medical diagnosis of psychosis, mental retardation, or neurological or cognitive visible impairments that could compromise their ability to participate in the study. All participants provided informed consent. Regarding aspects of drug use, the following data were collected: age at which drug use was initiated, age at which cocaine/crack was used, and the first drug used.

### 2.2 Instruments

#### 2.2.1 MINI International Neuropsychiatric Interview (MINI Version 5.0)

Data were collected through individual interviews with an average time span of sixty minutes, conducted by nursing and psychology post-graduate students and nurses trained for this purpose. To investigate the main psychiatric disorders, the MINI version 5.0 was used. MINI consists of a structured interview based on the Diagnostic and Statistical Manual 4º edition (DSM-IV-TR), and has a congruent diagnostic concordance with the Structured Clinical Interview for DSM-IV (SCID-IV) [22].

The MINI has demonstrated high specificity for each evaluated disorder and excellent inter-rater reliability [23]. The MINI suicidality total score has been previously used to measure suicide risk in clinical populations [24]. The MINI verifies the presence of the following psychiatric disorders: major and recurrent depressive episode, dysthymic disorder, suicide risk, hypo/manic episode, panic disorder, agoraphobia, social phobia, obsessive compulsive disorder, posttraumatic stress disorder, alcohol and substance addiction/abuse, psychotic syndrome, anorexia nervosa, bulimia nervosa, generalized anxiety disorder, and antisocial personality disorder. To determine the suicide risk, contributors responded to 11 questions measuring suicidal ideation, strategies, preparation, and attempt history. The risk of suicide is defined according to the final score: “low” (score 1-5), “moderate” (score 6-9) and “high”.

### 2.3 Genomic DNA Extraction and Genotyping

Genomic DNA was extracted from buccal epithelial cells following a modified protocol reported previously [25]. The concentration and integrity of the extracted samples were measured with the A260/280 ratio; a value of 1.8–2 was considered good quality and evaluated by spectrophotometry (Eppendorf^®^ AG, Hamburg, Germany). The qualitative and semi-quantitative evaluation of the samples was visualized in a 1% agarose gel electrophoresis stained with ethidium bromide using a transilluminator (Biotechnology-Locus). All samples were adjusted to a concentration of 10 ng/μl and distributed in a 96-well plate. The samples were kept at −20°C until analysis. IL-10 (-819C/T), TNFA (-308G/A) and ENOS (-786T/C) polymorphisms were amplified by Real-Time PCR using TaqMan assays (Applied Biosystem^®^, California, USA). Real-time PCR was performed in Step One Plus equipment (Applied Biosystem^®^, California, USA), under the following conditions: 60 ºC for 30 s, 95 Cº for 10 min, followed by 40 cycles of 95 ºC for 15 s and 60 ºC for 30 s; the data were analyzed by StepOnePlus software (Applied Biosystems^®^, California, USA).

### 2.4 Statistical Analyses

Statistical analyses were executed using SPSS version 22.0 (Statistical Package for the Social Sciences, IBM, Armonk, New York, USA). Normal distribution was tested using the Kolmogorov–Smirnov test and Shapiro–Wilk test. A descriptive analysis was carried out in the form of simple frequency, percentage, mean and standard deviation. Continuous data were expressed as mean and standard deviation and categorical as absolute frequency. Additionally, a binary logistic regression analysis based on the models described above was used to identify associations between the genetic profile and the presence or absence of psychiatric disorder. Genetics models (codominant, dominant, recessive and over dominant) were considered to evaluate the risk of mental disorders in cocaine and crack users associated with each SNP. The Hardy-Weinberg equilibrium (HWE) was verified using the chi-square test. Odds ratios (OR) and 95% confidence intervals (CI) were calculated considering OR<1 associated with protection and OR>1 associated with susceptibility/risk. The study power analysis was performed using the free software G * power version 3.0 with the following parameters: Chi-square tests and adequacy tests with contingency and Post-hoc evaluation tables. *P* values less than 0.05 were considered statistically significant.

## 3 RESULTS

### 3.1 Participant Characteristics

The majority of participants were male (86.8%), with a mean age ranging from 18 to 54 years old (M=31.4, SD=8.5). Regarding drug use, the mean age in years for starting drug use was 14.3 years (SD=4.3) and for starting using cocaine/crack was 20.3 years (SD=7.2). The highest proportion of the participants was self-reported, mixed color (54%, n=60), and with incomplete elementary school (62,6%). Marijuana was the first drug used by the majority (32.7%) of the participants, followed by alcohol use (31.7%). Regarding the diagnoses tracked throughMINI, the most prevalent psychiatric disorder was the current major depressive episode among the participants, and 30.4% had a recurrent major depressive episode. 100 (75%) of the participants had a current alcohol addiction, while 63 (56.3%) have experienced current alcohol abuse and 50 (44.6%) had generalized anxiety disorder, and 52 (46.4%) antisocial personality disorder. Among the subjects included in the study, 55 (49.1%) had suicide risk (Table **1**).

### 3.2 Effects of *IL-10* (-819C/T), *TNFA* (-30G/A8) and *ENOS* (-786T/C) Genotype and Allele on Mental Disorders and Suicide Risk

The genotype frequencies of the polymorphisms studied here were within the parameters of the Hardy-Weinberg Equilibrium (HWE), except the *ENOS* polymorphism (-T786C). Regarding the genotype frequency of this gene, it was possible to identify that the C/T genotype (41.2%) was the most frequent in both groups, while the T/T genotype (22.6%) was more prevalent in the case group compared to the control group. The C allele was the most frequent in both the study groups, however, the control group had a higher frequency (63%). The SNP-786 T/C of the *ENOS* gene in our study showed a higher frequency of the T/T genotype and the T allele in both the study groups (Table **2**).

Regarding the SNP -308 of the *TNFA* gene, the analysis of the genotypic and allele frequencies showed that the G/G genotype was more prevalent in all the investigated groups. None of the groups showed the A/A genotype. Concerning the allelic distribution of the *TNFA* polymorphism -308, the G allele was the most frequent in all groups. After performing the binary logistic regression, we compared cocaine and crack users with and without the respective psychiatric comorbidities according to the instruments used. When assessing the distribution between the genotype and allele frequencies of the case and control group, no significant difference was found. We identified the G/A genotype in the codominant model (OR = 0.24; CI = 0.06 - 0.87; p = 0.03) and the A allele (OR = 0.30; CI = 0.09 - 0.93; p = 0.03) to be associated with reduced risk for dysthymic disorder in our population.

In the same model, the A allele (OR = 0.24; CI = 0.08 - 0.72; p = 0.01) and the G/A genotype (OR = 0.18; CI = 0.05 - 0, 64; p = 0.008) were also correlated with protection for hypomanic episode (Table **3**). The T allele of -819C/T of IL-10 gene was associated with decreased risk of developing panic disorder (OR = 0.44; CI = 0.23 - 0.85; p = 0.01) (Table **4**), while the C allele was correlated with increased risk for alcohol addiction (OR = 1.97; CI = 1.00 - 3.88, p = 0.04) and alcohol abuse (OR = 1, 81; CI = 1.02 - 3.22; p = 0.04). The C allele of this polymorphism was also related to an increased risk for susceptibility to current psychotic syndrome (OR = 2.23; CI = 1.21 - 4.12; p = 0.01) and C/C genotype was correlated with increased odds of developing current psychotic syndrome in the codominant model (OR = 4.23; CI = 1.29 - 13.82; p = 0.01) and in the dominant model (OR = 3.07; CI = 1.32 - 7.14; p = 0.009) (Table **5**).

## 4 DISCUSSION

Our results describe for the first time a quantitative association of *IL-10* (-819C/T), *TNFA* (-308G/A) and *ENOS* (-786T/C) SNPs of candidate genes markers in cocaine/crack users with mental disorders in a Brazilian sample. These polymorphisms may affect the response to antidepressant therapy of major depressive disorder in the Polish population [26], high levels of anxiety in women after breast cancer surgery in the American population [27], methamphetamine-induced psychosis in the Japanese population [28], and schizophrenia in Germany population [29].

Cytokines have pro and anti-inflammatory properties and consist of a large group of small proteins that play a significant role in cell signaling and regulate a variety of processes in organisms, including proliferation and differentiation of many cells, mediation in defense response, and regulation of hematopoiesis [30]. In this context, the use of cocaine and crack has been shown to trigger immunomodulatory effects; the TNF alpha cytokine has been associated with the activation of the serotonergic system in patients with depression, suggesting a close relationship between cytokines and serotonergic systems. Few studies have linked the increased IL-10 serum levels [31] and decreased TNF alpha levels [18] with the development of psychiatric changes in drug users in the Brazilian and Spanish populations, respectively.

IL-10 is an anti-inflammatory cytokine activated by monocytes and lymphocytes and plays several immune functions, including a restraint biosynthesis of proinflammatory cytokines and suppression of cellular immunity in the central nervous system, favoring neural and glial cells [32]. In our population of cocaine and crack users, we identified T allele with a risk association for the development of the psychotic syndrome and alcohol abuse and C allele with a protective role for panic disorder. Elevated serum levels of IL-10 have been linked to susceptibility to schizophrenia in the Italian and Chinese population [33, 34]; however, decreased levels were found in patients with aggressive and psychotic behavior [35] and were found to be associated with decreased anti-inflammatory response in schizophrenia, especially in patients with severe symptoms in Chinese population [34]. Regarding panic disorder, there was a discrepancy in the serum levels of this cytokine in the Brazilian population [36], and IL-10 polymorphisms were not associated with panic disorder in an Estonian population [37]; however, elevated serum levels of IL-10 have been found in the American population [38].

TNF-α is a relevant pro-inflammatory cytokine that acts on the homeostasis of the immune system through the regulation of cell proliferation and death, as well as the response to infections and the inflammatory process. The *TNFA* -308G<A polymorphism has been evidenced by its role in susceptibility to various infectious, autoimmune and neurodegenerative diseases [39, 40]. The A allele was related to the high expression of the gene and consecutively to the increase in transcriptional activity [41, 42]. In our study, we identified the presence of the G/A genotype and the A allele to be correlated with decreased risk for the development of dysthymia and hypomanic episodes in cocaine and crack users. A study conducted in Arapiraca involving 165 patients with mental disorders and a history of attempted suicide concluded G/A genotype to be associated with a protective factor against the development of mental disorders [19].

Different results were found in studies evaluating the serum profile of this cytokine in dysthymia and hypomanic episodes. For example, in a population of dysthymic children and adolescents, reduced levels of plasma concentrations of TNF-α were identified when compared with those with major depression [43]. Furthermore, studies have identified TNF-α as an important biomarker of progression of bipolar disorder, with a significant increase in patients within a minimum of 10 years of diagnosis when compared to bipolar patients in the first episode of mood [44-46] and patients treated with mood stabilizers.

The effects of cocaine seem to be more prominent in the frontal cortical regions of the brain, including the prefrontal cortex [47], where cerebral blood flow is most affected [48, 49]. A previous study showed that the chronic administration of cocaine in mice induced cerebral ischemia [50]. Oxidative stress induces a decrease in nitric oxide levels, which leads to a disruption in muscle function due to the microvascular dysfunction in mental disorders, including depression [51, 52]. In our study, we did not identify a relationship between the ENOS (-786T/C) polymorphism and the development of mental disorders and suicide risk in cocaine and crack users; however, the power value of 11% for ENOS (-786T/C) polymorphism indicates that our sample size was inadequate and insufficient to detect a true association.

## 5 LIMITATION

The genetic background and miscegenation of the Brazilian population may clarify the different outcomes presented in this paper. Further, studies including a larger sample size genomewide association are needed to enhance the prediction of these results and better understand the interaction between genes and outcome of clinical mental disorders in cocaine/crack users.

## CONCLUSION

In conclusion, our results demonstrate the polymorphisms in *IL-10* (-819C/T) and *TNFA* (-308G/A) genes to be associated with the clinical phenotypes of mental disorders in cocaine/crack users. These results indicate that genetic polymorphisms of molecules involved in the synergic immune system might play an important role in the outcome of mental disorders in cocaine/crack users, and may be considered as prognostic biomarkers before stratification to improve addiction treatment. Furthermore, this study presents a significant step forward in determining the contribution of genetic factors in previously observed inter-individual variability with respect to mental disorders and addiction to cocaine/crack.

This investigation may help healthcare professionals to understand the pathophysiology of the disease, to identify prognosis, appropriate and individualized drug treatment, and perhaps formulate strategies to prevent pathologies of public health concern, especially in clinical practice, considering the mental health scenario of addicts, information regarding the genetic profile and their responses to drug treatment and coping at the psychosocial level.

## Figures and Tables

**Table 1:** Participant characteristics.

**Variable**	**n = 107**
Age (years), mean (SD)	31.4 (8.5)
Male sex, n (%)	86.8 (93)
Age at onset of drugs (years), mean (SD)	14.3 (4.3)
Age at onset of crack use (years), mean (SD)	20.3 (7.2)
**Race**	-
Mixed	54.1 (60)
White	20.6 (20)
Black	21.5 (23)
Yellow	3.7 (4)
**Educational level**	-
Illiterate	4.7 (5)
Complete high school education	10.3 (11)
Incomplete high school education	9.3 (10)
Complete elementary education	9.3 (10)
Incomplete elementary education	62.6 (67)
Complete higher education	1.9 (2)
Incomplete higher education	1.9 (2)
**The first drug used**	-
Marijuana	32.7 (35)
Alcohol	31.7 (34)
Tobacco/cigarette	8.1 (9)
Crack	3.7 (4)
Cocaine	2.8 (3)
Solvents	4.5 (5)
Others	16.5 (17)
Suicide risk	51.4 (55)
**Suicide risk level**	-
High	31.8 (34)
Moderate	3.7 (4)
Low	15.9 (17)
**Mental disorders**	-
Current major depressive episode	66.4 (71)
Recurrent major depressive episode	32.7 (35)
Dysthymic disorder	11.2 (12)
Current episode hypomanic	12.1 (13)
Past episode hypomanic	20.6 (22)
Lifetime panic disorder	24.3 (26)
Panic disorder with poor attack symptoms in lifetime	16 (15)
Current Panic Disorder	16.8 (18)
Panic disorder without current agoraphobia	14 (15)
Panic disorder with current agoraphobia	14 (15)
Agoraphobia	21.5 (23)
Social phobia	22.4 (24)
Obsessive-compulsive disorder	15 (16)
Post-Traumatic Stress Disorder	19.6 (21)
Alcohol addiction	76.6 (82)
Alcohol abuse	57.9 (62)
Current Psychotic Syndrome	37.4 (40)
Lifetime Psychotic Syndrome	27.1 (29)
Current mood disorder with psychotic features	10.3 (11)
Lifetime mood disorder with psychotic features	14 (15)
Bulimia nervosa	4.7 (5)
Generalized Anxiety Disorder	46.7 (50)
Antisocial personality disorder	47.7 (51)

**Table 2:** Genetic and allelic distribution of SNPs -819C/T (*IL-10*), -308G/A (*TNFA*) and -786T/C (*ENOS*) in cocaine and crack users and control group.

Gene/SNP	-	-	-	EHW*	G* Power
***IL-10 -819C/T***	**-**	**Case**	**Control**	***X*^2^ (p)**	**-**
Genotype	-	(n=102)	(n = 115)	-	*97%*
	C/C	37 (36.2)	45 (39.1)	0.99 (0.31)	-
	C/T	42 (41.2)	57 (47.9)	-	-
	T/T	23 (22.6)	15 (13)	-	-
Allele	-	-	-	-	-
-	C	116 (56.8)	145 (63)	-	-
-	T	88 (43.2)	87 (37)	-	-
*TNFA -308G/A*	-	-	-	-	-
Genotype	-	(n=107)	(n=107)	-	22%
-	G/G	85 (78.7)	88 (82.3)	2.51 (0.11)	-
-	G/A	23 (21.3)	19 (17.7)	-	-
Allele	-	-	-	-	-
-	G	278 (92.3)	195 (91.2)	-	-
-	A	23 (7.7)	19 (8.8)	-	-
*ENOS -786T/C*	-	-	-	-	-
Genotype	-	-	-	-	-
	C/C	10 (12.3)	15 (13.7)	6.06 (0.01)	11%
	T/T	48 (48.4)	50 (45.8)	-	-
	C/T	42 (39.3)	44 (40.5)	-	-
Allele	-	-	-	-	-
-	C	62 (31)	74 (33.9)	-	-
-	T	138 (69)	144 (66.1)	-	-

*Chi-square test. SNP - Single nucleotide polymorphism.

**Table 3:** Association of alleles and genetic models of SNPs -819C/T (*IL-10*), -308G/A (*TNFA*) and -786T/C (*ENOS*) with mood disorders and risk of suicide.

Current Major Depressive Episode			Recurrent Major Depressive Episode		Dysthymic Disorder		Manic Episode		Hypomanic Episode		Suicide Risk	
**Polymorphisms**	**-**		**-**		**-**		**-**		**-**		**-**	
*IL-10* -819C/T	OR with CI95%	p	OR with CI95%	p	OR with CI95%	p	OR with CI95%	p	OR with CI95%	p	OR with CI95%	p
Codominant	Reference	-	Reference	-	Reference	-	Reference	-	Reference	-	Reference	-
Dominant	0.88 (0.37 – 2.06)	0.88	0.67 (0.27 – 1.64)	0.38	0.86 (0.24 – 3.08)	0.82	1.21 (0.44 – 3.32)	0.69	0.48 (0.12 – 1.88)	0.29	0.81 (0.36 – 1.82)	0.61
Recessive	1.08 (0.40 – 2.89)	0.86	0.86 (0.32 – 2.31)	0.77	1.52 (0.30 – 7.49)	0.60	1.20 (0.36 – 4.04)	0.76	0.40 (0.11 – 1.39)	0.15	1.23 (0.48 – 3.13)	0.65
Overdominant	1.20 (0.51 – 2.78)	0.67	1.29 (0.56 – 2.99)	0.67	1.50 (0.44 – 5.01)	0.51	0.94 (0.34 – 2.55)	0.90	0.87 (0.26- 2.90)	0.83	1.42 (0.64 – 3.15)	0.38
Allele	0.85 (0.47 – 1.56)	0.61	0.82 (0.45- 1.49)	0.51	1.08 (0.45 – 2.61)	0.85	1.09 (0.53 – 2.23)	0.80	0.60 (0.26 – 1.37)	0.23	1.04 (0.59 – 1.83)	0.87
*TNFA* -308G/A	-	-	-	-	-	-	-	-	-	-	-	-
Codominant	1.55 (0.56 – 4.03)	0.39	0.60 (0.22 – 1.68)	0.33	0.24 (0.06 – 0.87)	0.03	0.96 (0.28 – 3.26)	0.95	0.18 (0.05 – 0.64)	0.008	0.55 (0.20 – 1.54)	0.26
Allele	1.48 (0.57 – 3.88)	0.41	0.64 (0.24 – 1.67)	0.36	0.30 (0.09 – 0.93)	0.03	0.96 (0.30 - 3.07)	0.95	0.24 (0.08 – 0.72)	0.01	0.58 (0.22 – 1.56)	0.28
*ENOS* -786T/C	-	-	-	-	-	-	-	-	-	-	-	-
Codominant	Reference	-	Reference	-	Reference	-	Reference	-	Reference	-	Reference	-
Dominant	1.28 (0.31 – 5. 32)	0.72	2.33 (0.62 – 8.72)	0.20	-	-	1.21 (0.44 – 3.32)	0.69	-	-	1.43 (0.37 – 5.43)	0.59
Recessive	1.15 (0.50 – 2.62)	0.73	0.64 (0.27 – 1.51)	0.31	2.40 (0.67 – 8.55)	0.17	1.20 (0.36 – 4.04)	0.76	-	-	0.85 (0.39 – 1.88)	0.70
Overdominant	0.79 (0.34 – 1.81)	0.58	1.11 (0.47 – 2.59)	0.80	0.64 (0.18 – 2.34)	0.51	0.94 (0.34 – 2.55)	0.90	-	-	1.02 (0.46 – 2.27)	0.94
Allele	1.03 (0.55 – 1.93)	0.92	0.64 (0.34 – 1.21)	0.17	2.45 (0.80 – 7.52)	0.11	2.27 (0.94 – 5.50)	0.06	1.40 (0.52 – 3.71)	0.50	0.84 (0.46 – 1.54)	0.84

*IL-10*: Interleukin– 10; *TNFA*: Tumor Necrosis Factor alpha; ENOS: Endothelial NOS; SNP - Single nucleotide polymorphism; OR- Odds ratio; CI: Confidential interval.

**Table 4:** Association of alleles and genetic models of SNPs -819C/T (*IL-10*), -308G/A (*TNFA*) and -786T/C (*ENOS*) with anxiety disorders.

-	Panic Disorder		Agoraphobia		Social Phobia		Obsessive-Compulsive Disorder			Posttraumatic Stress Disorder			Generalized Anxiety Disorder	
**Polymorphisms**	**OR with CI95%**	**p**	**OR with CI95%**	**p**	**OR with CI95%**	**p**	**OR with CI95%**	**p**	**OR with CI95%**		**p**	**OR with CI95%**		**p**
*IL-10* -819C/T	-	-	-	-	-	-	-	-	-		-	-		-
Codominant	Reference	-	Reference	-	Reference	-	Reference	-	Reference		-	Reference		-
Dominant	1.14 (0.40 – 3.26)	0.79	0.92 (0.34 – 2.34)	0.86	0.92 (0.34 – 2.43)	0.86	1.66 (0.55 – 5.02)	0.36	0.70 (0.24 – 2.02)		0.51	1.96 (0.86 – 4.46)		0.10
Recessive	0.37 (0.08 – 1.76)	0.21	0.94 (0.30 – 2.89)	0.91	0.94 (0.30 – 2.89)	0.91	1.30 (0.37 – 4.55)	0.68	2.21 (0.76 – 6.46)		0.14	0.69 (0.26 – 1.78)		0.44
Overdominant	1.54 (0.55 – 4.29)	0.40	1.13 (0.44 – 2.88)	0.79	1.13 (0.44 – 2.88)	0.79	0.46 (0.13 – 1.58)	0.22	0.72 (0.26 – 2.00)		0.53	0.68 (0.30 – 1.50)		0.34
Allele	0.44 (0.23 – 0.85)	0.01	0.89 (0.45 – 1.74)	0.74	1.00 (0.51 – 1.96)	0.98	0.96 (0.43 – 2.12)	0.92	0.74 (0.37 – 1.49)		0.40	1.50 (0.84 - 2.65)		0.16
*TNFA* -308G/A	-	-	-	-	-	-	-	-	-		-	-		-
Codominant	1.25 (0.37 – 4.16)	0.71	1.03 (0.30 – 3.47)	0.95	1.10 (0.32 – 3.70)	0.87	1.60 (0.33 – 7.75)	0.55	0.44 (0.14 – 1.35)		0.15	0.96 (0.35 – 2.61)		0.95
Allele	1.22 (0.38 – 3.86)	0.73	1.02 (0.32 – 3.26)	0.96	1.09 (0.34 – 3.46)	0.88	1.54 (0.33 – 7.03)	0.57	0.49 (0.17 – 1.37)		0.17	0.97 (0.37 – 2.49)		0.95
*ENOS* -786T/C	-	-	-	-	-	-	-	-	-		-	-		-
Codominant	Reference	-	Reference	-	Reference	-	Reference	-	Reference		-	Reference		-
Dominant	0.30(0.03-2.54)	0.27	1.60 (0.37 – 6.78)	0.52	1.50 (0.35 – 6.33)	0.58	0.65 (0.07 – 5.63)	0.70	1.98 (0.46 – 8.50)		0.35	1.87 (0.49 – 7.10)		0.35
Recessive	1.23(0.50-3.06)	0.64	1.77 (0.68 – 4.63)	0.24	1.96 (0.76 – 5.09)	0.16	1.53 (0.49 – 4.79)	0.46	0.42 (0.14 – 1.23)		0.11	1.60 (0.72 – 3.54)		0.24
Overdominant	1.11(0.44-2.78)	0.81	0.43 (0.15 – 1.23)	0.11	0.40 (0.14 – 1.12)	0.08	0.73 (0.22 – 2.37)	0.60	1.70 (0.62 – 4.64)		0.30	0.48 (0.21 – 1.09)		0.08
Allele	1.38 (0.67 – 2.83)	0.37	1.25 (0.59 – 2.64)	0.54	1.36 (0.65 – 2.85)	0.41	1.41 (0.56 – 3.51)	0.46	0.54 (0.26 – 1.13)		0.10	1.15 (0.63 – 2.11)		0.64

*IL-10*: Interleukin– 10; *TNFA*: Tumor Necrosis Factor alpha; ENOS: Endothelial NOS; SNP - Single nucleotide polymorphism; OR- Odds ratio; CI: Confidential interval.

**Table 5:** Allelic association and genetic models of SNPs -819C/T (*IL-10*), -308G/A (*TNFA*) and -786T/C (*ENOS*) with psychotic disorders, alcohol use and antisocial personality disorder.

**-**	**Current Psychotic Cyndrome**		**Mood Disorder with Psychotic Features**		**Alcohol Addiction**		**Alcohol Abuse**		**Antisocial Personality Disorder**	
**Polymorphisms**	**OR with CI95%**	***p***	**OR with CI95%**	***p***	**OR with CI95%**	***p***	**OR with CI95%**	***p***	**OR with CI95%**	***p***
IL-10 -819C/T	-	-	-	-	-	-	-	-	-	-
Codominant	Reference	-	Reference	-	Reference	-	Reference	-	Reference	-
Dominant	3.07 (1.32 – 7.14)	0.009	1.87 (0.50 – 6.96)	0.34	2.26 (0.76 – 6.76)	0.14	2.15 (0.91 – 5.07)	0.07	1.10 (0.49 – 2.48)	0.80
Recessive	2.58 (0.87 – 7.65)	0.087	0.35 (0.04 – 2.94)	0.33	0.72 (0.24 – 2.12)	0.55	0.56 (0.22 – 1.43)	0.22	0.83 (0.32 – 2.11)	0.69
Overdominant	0.62 (0.87 – 1.44)	0.27	0.94 (0.25 – 3.58)	0.93	0.63 (0.24 – 1.63)	0.34	0.75 (0.33 – 1.67)	0.48	1.03 (0.47 – 2.28)	0.92
Allele	2.23 (1.21 – 4.12)	0.01	2.81 (0.90 – 8.75)	0.07	1.97 (1.00 – 3.88)	0.04	1.81 (1.02 – 3.22)	0.04	1.14 (0.65 – 2.01)	0.63
TNFA -308G/A	-	-	-	-	-	-	-	-	-	-
Codominant	1.02 (0.36 – 2.87)	0.95	0.53 (0.12 – 2.23)	0.38	0.33 (0.07 – 1.55)	0.16	0.76 (0.27 – 2.13)	0.61	1.01 (0.37 – 2.37)	0.97
Allele	1.02 (0.38 – 2.72)	0.95	0.57 (0.15 – 2.15)	0.41	0.36 (0.08 – 1.61)	0.18	0.78 (0.29 – 2.08)	0.63	1.01 (0.39 – 2.60)	0.97
ENOS -786T/C	-	-	-	-	-	-	-	-	-	-
Codominant	Reference	-	Reference	-	Reference	-	Reference	-	Reference	-
Dominant	1.72 (0.46 – 6.41)	0.41	2.25 (0.41 – 12.2)	0.34	1.14 (0.22 – 5.81)	0.87	0.66 (0.18 – 2.46)	0.54	1.79 (0.47 – 6.78)	0.39
Recessive	1.13 (0.50 – 2.55)	0.75	1.34 (0.38 – 4.72)	0.64	1.84 (0.69 – 4.88)	0.22	1.32 (0.59 – 2.94)	0.49	0.56 (0.25 – 1.24)	0.15
Overdominant	0.70 (0.31 – 1.62)	0.41	0.48 (0.12 – 1.93)	0.30	0.52 (0.20 – 1.35)	0.18	0.87 (0.39 – 1.96)	0.74	1.45 (0.65 – 3.22)	0.36
Allele	0.86 (0.34 – 2.80)	0.89	0.95 (0.37 – 2.48)	0.93	1.36 (0.67 – 2.76)	0.38	1.28 (0.69 – 2.34)	0.42	0.63 (0.34 – 1.15)	0.13

*IL-10*: Interleukin– 10; *TNFA*: Tumor Necrosis Factor alpha; ENOS: Endothelial NOS; SNP - Single nucleotide polymorphism; OR- Odds ratio; CI: Confidential interval.

## Data Availability

The data supporting the findings of the article are available in the Repositório da Universidade Federal de Alagoas at http://www.repositorio.ufal.br/.
